# Genetic Markers Associated to Dyslipidemia in HIV-Infected Individuals on HAART

**DOI:** 10.1155/2013/608415

**Published:** 2013-09-26

**Authors:** Rosmeri K. Lazzaretti, Aline S. Gasparotto, Marina G. de M. Sassi, Carísi A. Polanczyk, Regina Kuhmmer, Jussara M. Silveira, Rossana P. Basso, Cezar A. T. Pinheiro, Mariângela F. Silveira, Eduardo Sprinz, Vanessa S. Mattevi

**Affiliations:** ^1^Serviço de Cardiologia, Hospital de Clínicas de Porto Alegre, Universidade Federal do Rio Grande do Sul, 90035-903 Porto Alegre, RS, Brazil; ^2^Universidade Federal de Ciências da Saúde de Porto Alegre, Rua Sarmento Leite 245, Sala 309, 90050-170 Porto Alegre, RS, Brazil; ^3^Serviço de Cardiologia, Hospital de Clínicas de Porto Alegre, Universidade Federal do Rio Grande do Sul, Instituto de Avaliação de Tecnologias em Saúde, 90035-903 Porto Alegre, RS, Brazil; ^4^Hospital Universitário Dr. Miguel Riet Correa Jr., 96200-000 Rio Grande, RS, Brazil; ^5^Serviço de Assistência Especializada em HIV/AIDS, Universidade Federal de Pelotas, 96030-001 Pelotas, RS, Brazil; ^6^Serviço de Medicina Interna, Hospital de Clínicas de Porto Alegre, Universidade Federal do Rio Grande do Sul, 90035-903 Porto Alegre, RS, Brazil

## Abstract

This study evaluated the impact of 9 single nucleotide polymorphisms (SNPs) in 6 candidate genes (*APOB, APOA5, APOE, APOC3, SCAP*, and *LDLR*) over dyslipidemia in HIV-infected patients on stable antiretroviral therapy (ART) with undetectable viral loads. Blood samples were collected from 614 patients at reference services in the cities of Porto Alegre, Pelotas, and Rio Grande in Brazil. The SNPs were genotyped by conventional polymerase chain reaction (PCR) and real-time PCR. The prevalence of dyslipidemia was particularly high among the protease inhibitors-treated patients (79%). *APOE* (rs429358 and rs7412) genotypes and *APOA5* −1131T>C (rs662799) were associated with plasma triglycerides (TG) and low-density-lipoprotein cholesterol levels (LDL-C). The *APOA5* −1131T>C (rs662799) and *SCAP* 2386A>G (rs12487736) polymorphisms were significantly associated with high-density-lipoprotein cholesterol levels. The mean values of the total cholesterol and LDL-C levels were associated with both the *APOB* SP *Ins/Del* (rs17240441) and *APOB XbaI* (rs693) polymorphisms. In conclusion, our data support the importance of genetic factors in the determination of lipid levels in HIV-infected individuals. Due to the relatively high number of carriers of these risk variants, studies to verify treatment implications of genotyping before HAART initiation may be advisable to guide the selection of an appropriate antiretroviral therapy regimen.

## 1. Introduction

The use of antiretroviral therapy (ART) as a standard of care has changed the prognosis of human immunodeficiency virus (HIV) infection by decreasing mortality and improving quality of life [1, 2]. Despite the clinical benefits, long-term ART is associated with a complex spectrum of unwanted metabolic effects, including dyslipidemia that eventually might lead to increased risk of cardiovascular diseases [3].

Nevertheless, these side effects are not universal to all individuals on ART and even vary in individuals with comparable ART, demographic, immunologic, and virological characteristics. This variability suggests that host genetic factors and inherited predispositions may have a significant influence on the appearance of metabolic alterations [4].

The exact mechanism of dyslipidemia is not fully understood but is most likely multifactorial. In the general population, genetic variation accounts for approximately 43%–83% of the variability in lipid plasma levels [5]. Recent candidate gene studies [6–11] as well as genome-wide-based association studies have identified certain single nucleotide polymorphisms (SNPs) that could account for a significant portion of the variation in blood lipid levels [12–14]. 

In HIV infection, genetic predisposition may help to explain the variability among patients with respect to the effects of protease inhibitors (PIs) on lipid metabolism [10, 11]. We have hypothesized that this variation is attributable to the joint effect of HIV infection and ART together with the underlying genetic predisposition present in these individuals. The aim of this study was to investigate the frequencies of 9 SNPs in 6 candidate genes and to identify associations between these SNPs and the plasma lipid levels of patients on stable ART with undetectable viral loads.

## 2. Methods

### 2.1. Subjects

We conducted a cross-sectional study with 614 patients who were diagnosed with HIV-1 infection according to the criteria of the Centers for Disease Control and Prevention [15]. All subjects were more than 17 years old, had regularly used ART for at least 12 months, had a viral load below the detection limit of the test (50 copies/mL; Versant HIV-1 RNA 3.0 Assay (bDNA), Siemens, Germany), and were recruited from three referral centers in southern Brazil (HIV/AIDS Ambulatory Unit of Hospital de Clínicas from Porto Alegre/RS, HIV Ambulatory Care of Hospital Universitário Dr. Miguel Riet Correa Jr. from Rio Grande/RS, and HIV/AIDS Specialized Assistance Service from Pelotas/RS) from March 2006 to November 2008. Pregnant women and those with neurological disease that prevented understanding and proper consent were not included in the study. The study protocol was approved by the Research Ethics Committees of the three centers and of the Universidade Federal de Ciências da Saúde de Porto Alegre, and all participants signed an informed consent statement before they were included in the study (protocol numbers: 05/295, 718/08, 154/07, and 141/06, resp.).

### 2.2. Study Protocol

The routine evaluation consisted of visits every 4 months in each center for an evaluation by the patients' attending physicians as well as laboratory evaluations that included measurements of CD4 cell counts, viral load, and lipid levels. The patients were invited to participate in the study and had their information and a blood sample for DNA extraction collected during one of these visits. 

An interview was performed at enrollment to obtain demographic and lifestyle information. Details of HIV infection (time from diagnosis as well as current and prior antiretroviral medications), lipid-lowering intervention, and relevant clinical variables were obtained from medical records. The interviewer phenotypically defined the patients' ethnicities because there might be a strong cultural tendency to claim European ancestry in Brazil [16]. Patients were classified as Euro- or Afro-descendants because the Amerindian contribution is very low in the Brazilian South Region [17].

### 2.3. Laboratory Analysis

Blood samples were collected and sent to the Molecular Biology Laboratory for DNA extraction. Lipid profiles included determinations of total cholesterol (TC), high-density lipoprotein (HDL-C), triglycerides (TG), and, when possible, low-density lipoprotein (LDL-C) after fasting for 12 hours. LDL-C was calculated using the Friedewald formula, LDL-C = TC − HDL-C − TG/5, if triglyceride levels were below 400 mg/dL. 

Dyslipidemia was defined by fasting triglycerides plasma levels ≥150 mg/dL and/or fasting total cholesterol ≥200 mg/dL and/or LDL-C ≥130 mg/dL and/or HDL-C <40 mg/dL. Participants were instructed not to perform any vigorous physical activity or ingest alcohol in the 24 hours prior to the blood collection [18].

Genomic DNA was obtained from peripheral leukocytes by a standard salting-out technique [19]. The genotypes of apolipoprotein B gene (*APOB*) polymorphisms were determined using polymerase chain reaction (PCR) based procedures in 410 individuals from the outpatient clinic of the Hospital de Clínicas de Porto Alegre. The polymorphism of insertion/deletion of signal peptide SP *Ins/Del* (rs17240441) was amplified by PCR using primers as previously described [20] and directly analyzed by electrophoresis in 8% polyacrylamide gels. The *Xba*I, 7673C>T (rs693) polymorphism was amplified by PCR using the primers described by Pan et al. [21], and genotypes were determined by digestion with *Xba*I restriction endonuclease and electrophoresis in 2% agarose gels. 

The SNPs of apolipoprotein A-V (*APOA5*) *−*1131T>C (rs662799) and S19W (56C>G; rs3135506), apolipoproteinE (*APOE*) 334T>C (E4; rs429358) and 472C>T (E2; rs7412), apolipoprotein C-III (*APOC3*) 3238C>G (rs5128), sterol regulatory element-binding factor cleavage-activating protein (*SCAP*) 2386A>G (rs12487736), and low-density lipoprotein receptor (*LDLR*) intron 19G>T (rs6511720) were genotyped by real-time PCR using the TaqMan methodology and are listed in Table 1. Candidate SNPs were selected via review of PubMed reports of SNP associations with dyslipidemia in the general population or among HIV-infected individuals [6–11].

### 2.4. Statistical Analyses

The results are expressed as the mean ± standard deviation (SD) for continuous variables and as proportions for categorical variables. Variables that did not have a normal distribution (triglycerides) were transformed into natural logarithms before the statistical tests were applied. Allele frequencies were estimated by gene counting. *χ* ^2^ analysis that was used to test for deviations in genotype frequencies from the Hardy-Weinberg equilibrium (HWE).

For the *APOC3* 3238C>G (rs5128), *APOA5 −*1131T>C (rs662799) and S19W (56C>G) (rs3135506)*, SCAP *2386A>G (rs12487736), and* LDLR* intron 19G>T (rs6511720) polymorphisms, the association analyses were performed according to the dominant model due to the low number of individuals who were homozygous for the minor allele and were pooled with subjects with the heterozygous genotype. As for *APOE* SNPs 334T>C (rs429358) and 472C>T (rs7412), which together define the *APOE* E2, E3, and E4 alleles, the subjects were analyzed in 3 genotype categories: E2/E3, homozygotes for the E3 allele and E3/E4. Subjects with the rare genotypes E2/E2 (*n* = 5), E2/E4 (*n* = 9), and E4/E4 (*n* = 16) were excluded from the statistical analyses.

The Testing Haplotype Effects in Association Studies Program (version 3.1, THESIAS, Paris, France) was used for the analysis of linkage disequilibrium between polymorphisms within the same gene [22].

General linear model analyses were used to test for the interaction between SNPs and variables and to adjust the lipid profile for covariables. The following variables were included in the models and underwent stepwise removal according to the greatest *P* values: ethnic group, gender, age, physical activity, cigarette smoking, use of lipid-lowering agents, PI use, body mass index (BMI), and the presence of polymorphism. Only those variables that were significant predictors were included in the final model. Correction for multiple testing was performed using the Bonferroni method. The data were analyzed with the Statistical Package for Social Sciences (version 20.0, SPSS, Chicago, Illinois). The value indicating statistical significance was *P* < 0.05.

## 3. Results

### 3.1. Study Participants

The main demographic, clinical, and metabolic characteristics of the individuals enrolled in the study are shown in Table 2. Of the 614 patients, 55.5% were males, 57% were characterized as Euro-descendants, and the mean patient age was 43 ± 10 years. The mean duration of ART was 68 ± 41 months. Regarding metabolic parameters, 245 (41%) patients had hypercholesterolemia, 293 (49%) had hypertriglyceridemia, and 185 (31%) had low HDL-C levels. 

The total patient population was stratified according to current PI use or nonuse. The prevalence of dyslipidemia was particularly high among the PI-treated patients (79% versus 69% in nonusers; *P* = 0.006). Although the mean levels of TC and LDL-C did not differ significantly between the PI-treated and non-PI-treated subjects, the latter had higher plasma HDL-C levels (51 ± 17 mg/dL versus 46 ± 14 mg/dL; *P* < 0.001) and lower triglycerides levels (170 ± 131 mg/dL versus 223 ± 202 mg/dL, *P* < 0.001) than the PI-treated subjects. Therefore, PI use was tested as a covariate in all statistical analyses and was included when significant. 

The genotype and allele frequencies of the analyzed SNPs are shown in Table 1. For all studied polymorphisms, there was no departure from Hardy-Weinberg equilibrium, except for *APOC3* 3238C>G (rs5128).  Table 3 summarizes the association analysis of the SNPs with serum lipid concentrations. 

### 3.2. Triglycerides

Two SNPs contributed significantly to the modification of TG levels. The plasma TG levels were different among *APOE* genotypes (*P* = 0.033), the E2 allele being associated with increased TG levels (Tukey test, E2/E3 versus E3/E3, *P* = 0.003; E2/E3 versus E3/E4, *P* = 0.029). However, this result was no longer significant after correction for multiple testing (*P*_corrected_ = 0.132).

The effect of the *APOA5 −*1131T>C (rs662799) polymorphism on plasma lipids was observed in C-allele carriers, who presented higher triglyceride levels than those with the TT genotype (243 ± 230 mg/dL and 187 ± 157 mg/dL, respectively; *P* = 0.003). This nominal *P*-value remained statistically significant after Bonferroni correction for multiple comparisons (*P*_corrected_ = 0.024).

### 3.3. HDL Cholesterol

The *APOA5 −*1131T>C (rs662799) polymorphism was also significantly associated with HDL-C levels. The TT homozygotes presented higher HDL-C concentrations than C-carriers (*P* = 0.047) after the same statistical approach and adjustment for covariates. Furthermore, a statistically significant association of the *SCAP* 2386A>G (rs12487736) variant was observed with HDL-C levels (*P* = 0.032). As shown in Table 3, the 2386GG homozygotes had lower HDL-C levels, while the 2386AA homozygotes showed an increase of 5 ± 2 mg/dL when compared to GG homozygotes (Tukey test, AA versus GG, *P* = 0.014). However, both associations were no longer significant after the Bonferroni correction for multiple comparisons.

### 3.4. Total Cholesterol and LDL Cholesterol

Initially, the average TC levels were associated with both the *APOB* SP *Ins/Del* (rs17240441; Table 3, *P* = 0.036) and *APOB Xba*I, 7673C>T (rs693; *P* = 0.007) polymorphisms. The *post hoc* test showed that there were differences between the homozygotes for both polymorphisms (Tukey test, *Ins/Ins* versus *Del/Del*, *P* = 0.027 and C/C versus T/T, *P* = 0.007, resp.). Neither results were significant after Bonferroni correction was applied (*P*_corrected_ = 0.320 and *P*_corrected_ = 0.056, resp.).

The mean LDL-C levels were also different between the genotypes (*P* = 0.007 and *P* = 0.002, resp.) for both polymorphisms and were statistically significant after Bonferroni correction was applied (*P*_corrected_ = 0.048 and *P*_corrected_ = 0.016, resp.). The Tukey test showed that, in the polymorphism of the signal peptide, differences were found between the homozygotes (*Ins/Ins *versus *Del/Del*, *P* = 0.006) and in the polymorphism in exon 26 of the gene the homozygous group with regard to the C allele differed from the other groups (C/C versus C/T, *P* = 0.006; C/C versus T/T, *P* = 0.008). 

A significant linkage disequilibrium (*D*′ = 0.72, *P* < 0.001) was detected between polymorphisms associating alleles *Del* and T, as well as *Ins *and C. Based on these results, we analyzed the effect of the haplotype combining alleles *Del *and T on the levels of these lipid parameters. The haplotype analysis showed that homozygotes for the risk haplotype showed significantly higher mean levels of TC and LDL-C (Table 4). The *post hoc* test showed that the differences were found between noncarriers and homozygotes for the *Del* T haplotype (Tukey test, *P* = 0.036 and *P* = 0.038, resp.). 

The *APOE *genotype was associated with elevated plasma LDL-C levels (*P* = 0.002; *P*_corrected_ = 0.008; Table 3). In contrast to what was observed regarding the TG levels, the E2 allele was associated with a decrease in the LDL-C levels, while the E4 allele was associated with an increase, as the differences were found between the E2 and E4 allele carriers (Tukey test, E2/E3 versus E3/E3, *P* = 0.011; E2/E3 versus E3/E4, *P* = 0.005). 

Regarding *APOA5* S19W (56C>G) (rs3135506), there was a marginal association with LDL-C levels. Carriers of the G allele and C/C genotype showed a mean and an SD of 112 ± 35 mg/dL and 106 ± 37 mg/dL, respectively (*P* = 0.047), that was adjusted for gender, age, BMI, and use of lipid-lowering agents. However, it did not remain significant after Bonferroni correction.

No significant contribution to plasma TC, TG, HDL-C, or LDL-C was identified in the present dataset with regard to *APOC3* 3238C>G (rs5128) and *LDLR* intron 19G>T (rs6511720). 

Analyses were also performed separately for users and nonusers of IPs (data not shown), and similar effects were observed in both groups, although in some cases these effects were not significant. 

## 4. Discussion

The contributions of several polymorphisms to dyslipidemia in 614 HIV-1-infected patients on HAART were addressed in the present multicenter study. Nine polymorphisms in 6 candidate genes were analyzed for their association with dyslipidemia. As expected according to the literature, this adverse effect was more prevalent in patients receiving PIs ([23] and references therein). However, we found no statistically significant difference in TC and LDL-C levels between PI-treated and non-PI-treated patients, probably associated with the more prevalent use of lipid-lowering agents by the PI group (21 versus 14%, *P* = 0.021).

The allelic frequencies of the* APOB*, *APOA5*, *APOE*, *SCAP,* and *LDLR* genotypes were similar to those previously described in populations from the same geographical region or ethnicity [8–11]. The *APOC3* 3238C>G (rs5128) genotypic frequencies were not distributed according to what was expected under Hardy-Weinberg equilibrium (*P* = 0.032; Table 1). This small deviation was most likely due to the lower number of homozygotes (2 patients, 0.3%) for the rare allele observed in our study; 7 were expected according to Hardy-Weinberg's proportions. Nevertheless, the allele frequencies (*G* = 0.11) were very close to those found in previous studies of individuals not infected with HIV who live in Southern Brazil (*G* = 0.10), as shown by Fiegenbaum et al. [8], and to participants in the Swiss HIV Cohort Study (SHCS), which were *G* = 0.10 and *G* = 0.09, as shown by Rotger et al. [11] and Tarr et al. [24], respectively. 

Our study demonstrated a strong association between the E3/E4 genotype and elevated plasma LDL-C, which is in agreement with the results of previous studies [25, 26]. The E4 variant is typically associated with increased levels of LDL-C and low plasma TG levels, whereas the E2 variant is associated with lower LDL-C levels in the general population [25, 26]. In a large meta-analysis, the E4 variant was associated with a 42% increase in cardiovascular disease risk [27]. Although E2 isoforms bind to LDL receptors much more weakly than E3 or E4 isoforms, the catabolism of the particle containing isoform E2 is slower; this may result in a lower rate of LDL-C formation. Moreover, according to the conclusion of the meta-analysis of Bennet et al. [28], there is an approximately linear relationship between the *APOE* genotypes and both LDL-C levels and to the risk of cardiovascular disease. 

Carriers of non-E3/E3 genotypes of *APOE *appear to be at risk of ritonavir-associated hypertriglyceridemia, and this risk appears to be enhanced by the association with *APOC3 *variants [29]. According to the SHCS, the interaction between *APOE* and *APOC3* is associated with an extreme risk of developing hypertriglyceridemia in individuals treated with ritonavir [24]. Our data did not allow us to perform the same analyses, as the vast majority of our PI-users were on ritonavir. Nevertheless, our findings are in agreement with these data reinforcing the association of E2 allele and increased TG levels. With respect to the E3/E3 genotype, according to our data as well as to those of [30], HIV-positive patients have a lower risk of developing high triglyceride levels after starting HAART than non-E3/E3 genotypes. 

*APOA5* also plays an important role in the modulation of blood lipid metabolism; it is predominantly synthesized in the liver and is secreted into the plasma, where it plays a central role in regulating TG metabolism [31, 32]. Two polymorphisms in the *APOA5* gene, −1131T>C and S19W (56C>G), have been shown to be associated with elevated triglyceride levels in different populations [31, 33]. In our study, for *APOA5* −1131T>C, individuals with at least one C allele had higher TG levels and lower levels of HDL-C. However, only the association with TG levels remained significant after the Bonferroni correction was applied. These results are consistent with previous study in HIV-infected patients, indicated that −1131C carriers experienced marked increases in triglyceride levels during a 3-year follow-up, while no change was recorded in patients carrying the −1131T normal allele [34]. A similar effect, but of lesser magnitude, was demonstrated regarding changes in cholesterol [34]. 

To our knowledge, this is the first study conducted in an HIV-positive setting to assess the association of *SCAP* 2386A>G rs12487736 with dyslipidemia. The SCAP pathway controls cellular cholesterol homeostasis. Initially, in our analysis, we found that AA homozygous patients showed higher HDL-C levels; however, this result was no longer significant after Bonferroni correction was applied. Carriers of the 2386G allele from non-HIV-infected Brazilians treated with simvastatin exhibit reduced TC and TG levels, as shown by Fiegenbaum et al. [7], but no association was found with HDL-C levels. 

The mean TC and LDL-C levels showed statistically significant differences for genotypes of both polymorphisms in the *APOB *gene: SP *Ins/Del* (rs17240441) and *Xba*I, 7673C>T (rs693). The homozygotes for the alleles T and *Del* had higher levels of these lipids than individuals heterozygous for both alleles, who had intermediate levels; this result is compatible with a co-dominant effect of these polymorphisms. These results are in agreement with published data showing that the *Del* and T alleles are associated with increased levels of TC and/or LDL-C in different populations with distinct diseases [9, 35–37]. However, Xu et al. and Ye et al. found no association of the polymorphism in the signal peptide of the gene with TC and LDL-C levels in the Finnish and Chinese populations, respectively [38, 39].

The significant linkage disequilibrium observed between polymorphisms, as reported in other studies [20, 39], led to an analysis of risk haplotype (patients with a combination of alleles *Del *and T). The patients who were homozygous for the risk haplotype showed higher total cholesterol and LDL-C, while an intermediate effect was observed in patients with the heterozygous haplotype. Rios et al. [9] also observed the association of the haplotype with LDL-C levels in a Brazilian population in a comparison of patients with and without coronary artery disease. 

There are two possible causes for these changes in lipid metabolism related to both polymorphisms in the *APOB* gene. The first cause is that three amino acids, leu-ala-leu, that are included in the allele* Ins* and deleted in the allele *Del* could alter the hydrophobicity of the signal peptide of the protein and, thereby, alter the rate of translocation of new *APOB *peptides synthesized in the cytoplasm to the endoplasmic reticulum [38]. The second hypothesis is based on the fact that there is no amino acid change in the functional protein in both polymorphisms. These could then be in linkage disequilibrium with an unknown change in the DNA that may cause lipid abnormalities [20].

Two further SNPs that were proposed in the literature (*APOC3* 3238C>G (rs5128) and *LDLR* intron 19G>T (rs6511720) did not contribute to the plasma lipid levels in the present dataset, which may reflect a limited effect of these SNPs in HIV-infected patients. 

All analyses were also performed by stratifying the patients according to PI use (data not shown); however, similar effects were observed in both groups. Furthermore, the variables were not statistically significant in some analyses, and this was most likely attributable to the reduction in the number of individuals when they are analyzed separately. This finding suggests that in our sample, the lipid-gene interaction is independent of the type of HAART used and that the variation observed is similar to that found in the general population.

We acknowledge some limitations of the present study. The cross-sectional design and the inclusion of patients receiving several different antiretroviral regimens, which is a difficulty inherent to any study involving current HAART, as by definition should include at least three different drugs. This fact might hinder the accomplishment of any pharmacogenomics study regarding this therapy. Furthermore, we found a deviation from the HWE observed in the current population for the *APOC3* 3238C>G (rs5128) SNP. Although this deviation may indicate genotyping error, laboratory procedures were in place to detect errors, including blinded, no-template controls and DNA sample replicates. Another limitation that we might acknowledge is the restricted number of SNPs analyzed. We cannot rule out the possibility that other SNPs in the genes investigated here are associated with the phenotypes studied. 

In conclusion, our data support the importance of genetic factors on of lipid levels in HIV-infected individuals from a previously uninvestigated region of the world. We found no evidence of interaction of these genetic variants with the use of non-nucleoside transcriptase reverse inhibitors or protease inhibitors. Due to the relatively high number of carriers of these risk variants (e.g., *APOA5 *−1131T>C = 15%, *APOB* risk haplotype = 47%, *APOE* = 20.1%), studies to verify treatment implications of genotyping are desirable. 

## Figures and Tables

**Table 1:** Genotypic and allelic frequencies of polymorphisms analyzed.

Polymorphisms	Genotypic frequency	Allelic frequency		
*APOA5* −1131T>C (rs662799)^a^		T	C	
T/T	515 (84.7)	0.92	0.08	
T/C	90 (14.8)			
C/C	3 (0.5)			
Total	**608**			
*APOA5* S19W (56C>G; rs3135506)^a^		C	G	
C/C	500 (82)	0.90	0.10	
C/G	103 (16.9)			
G/G	7 (1.1)			
Total	**610**			
*APOB* SP *Ins/Del* (rs17240441)^a^		*Ins *	*Del *	
*Ins/ins *	193 (47.3)	0.70	0.30	
*Ins/del *	176 (43.1)			
*Del/del *	39 (9.6)			
Total	**408**			
*APOB XbaI*, 7673C>T (rs693)^a^		C	T	
C/C	122 (30)	0.55	0.45	
C/T	203 (49.9)			
T/T	82 (20.1)			
Total	**407**			
*APOC3* 3238C>G (rs5128)^b^		C	G	
C/C	478 (78.8)	0.89	0.11	
C/G	127 (20.9)			
G/G	2 (0.3)			
Total	**607**			
*APOE* ^ a^		E2	E3	E4
E2/E2	5 (0.8)	0.07	0.79	0.14
E2/E3	67 (11.2)			
E2/E4	9 (1.5)			
E3/E3	379 (63.6)			
E3/E4	120 (20.1)			
E4/E4	16 (2.7)			
Total	**596**			
*LDLR* intron 19G>T (rs6511720)^a^		G	T	
G/G	487 (80.2)	0.90	0.10	
G/T	113 (18.6)			
T/T	7 (1.2)			
Total	**607**			
*SCAP* 2386A>G (rs12487736)^a^		A	G	
A/A	168 (27.7)	0.52	0.48	
A/G	296 (48.9)			
G/G	142 (23.4)			
Total	**606**			

The difference in the number of individuals among single nucleotide polymorphisms (SNPs) is due to failure in genotyping some SNPs in the whole sample.

Genotypic frequencies presented as number of patients (%).

^a^ *χ*² test for Hardy-Weinberg equilibrium; *P* > 0.05.

^b^ *χ*² test for Hardy-Weinberg equilibrium; *P* = 0.032.

**Table 2:** Characteristics of the study participants.

	All study participants *n* = 614	PI-sparing ART *n* = 311	PI-based ART *n* = 303	*P* value
Demographic				
Age, years	43.0 ± 10	42 ± 10	43 ± 9	0.114^§^
Male sex, *n* (%)	341 (55.5)	179 (58)	162 (54)	0.348*
Ethnicity, *n* (%)				
Euro-Brazilians	349 (57)	166 (48)	183 (60)	0.094*
Afro-Brazilians	265 (43)	145 (47)	120 (40)	
Physical activity, *n* (%)	156 (25)	74 (24)	82 (27)	0.389*
Cigarette smoking, *n* (%)	186 (30)	103 (33)	83 (28)	0.153*
Clinical				
CD4, cells/uL	533 ± 266	516 ± 255	552 ± 277	0.095^§^
Therapy time (months)	68 ± 41	57 ± 38	76 ± 41	<0.001^§*¥*^
Lipid-lowering drugs, *n* (%)	105 (17)	42 (14)	63 (21)	0.021*
Metabolic				
Triglycerides, mg/dL	196 ± 171	170 ± 131	223 ± 202	<0.001^¶*¥*^
Total cholesterol, mg/dL	192 ± 48	189 ± 44	196 ± 52	0.056^§^
HDL-C, mg/dL	48 ± 16	51 ± 17	46 ± 14	<0.001^§^
LDL-C, mg/dL	107 ± 37	105 ± 34	109 ± 40	0.175^§^
Dyslipidemia, *n* (%)	455 (74)	215 (69)	240 (79)	0.006*
Hypertriglyceridemia	293 (49)	122 (40)	171 (59)	<0.001*
Hypercholesterolemia	245 (41)	118 (39)	127 (44)	0.267*
Low HDL-C	185 (31)	74 (24)	111 (38)	<0.001*

Data presented as mean ± SD or number of patients (%).

PI: protease inhibitors; ART: antiretroviral therapy; HDL-C: high-density lipoprotein; LDL-C: low-density lipoprotein.

^¥^ *P* value expressed with tests performed with ln-transformed variable.

^§^Student *t*-test for independent samples.

*χ ^2^-Test with Yates correction.

^¶^Student *t*-test for independent samples with ln-transformed variable.

**Table 3:** Mean metabolic variables according to polymorphisms analyzed.

Polymorphisms	Total cholesterol (mg/dL)		LDL-C (mg/dL)		HDL-C (mg/dL)		Triglycerides (mg/dL)	
	*N*	M ± SD	*N*	M ± SD	*N*	M ± SD	*N*	M ± SD
*APOA5* −1131T>C (rs662799)								
T/T	499	191 ± 80	458	106 ± 72	498	48 ± 88	499	187 ± 157
T/C + C/C	92	196 ± 78	83	107 ± 12	92	45 ± 16	92	243 ± 230
*P* value		0.549		0.953		0.047^$^		0.003^∗*£*^
*P* value corrected**		1.000		1.000		0.376		0.024
*APOA5* S19W (56C>G; rs3135506)								
C/C	486	192 ± 49	443	106 ± 37	485	48 ± 15	486	199 ± 177
C/G + G/G	106	197 ± 43	99	112 ± 35	107	50 ± 17	107	183 ± 144
*P* value		0.330		0.047^#^		0.130		0.398*
*P* value corrected**		1.000		0.376		1.000		1.000
*APOB* SP *Ins/Del* (rs17240441)								
* Ins/ins *	192	189 ± 42^a^	176	99 ± 31^c^	192	52 ± 15	193	195 ± 167
* Ins/del *	175	193 ± 51^ab^	158	106 ± 36^cd^	175	50 ± 14	176	195 ± 193
* Del/del *	39	210 ± 45^b^	37	120 ± 41^d^	39	54 ± 13	39	187 ± 103
*P* value		0.036^§^		0.006^§^		0.245		0.996*
*P* value corrected**		0.288		0.048		1.000		1.000
*APOB XbaI*, 7673C>T (rs693)								
C/C	122	183 ± 42^e^	112	95 ± 35^g^	122	52 ± 15	122	187 ± 126
C/T	202	194 ± 42^ef^	185	107 ± 31^h^	201	50 ± 14	203	193 ± 163
T/T	81	203 ± 59^f^	73	110 ± 41^ih^	82	51 ± 12	82	212 ± 248
*P* value		0.007^§^		0.002^§^		0.705		0.791*
*P* value corrected**		0.056		0.016		1.000		1.000
*APOC3* 3238C>G (rs5128)								
C/C	464	193 ± 48	424	107 ± 37	465	49 ± 16	440	191 ± 162
C/G + G/G	125	192 ± 49	115	108 ± 39	124	47 ± 15	124	210 ± 214
* P* value		0.718		0.833		0.295		0.905*
* P* value corrected**		1.000		1.000		1.000		1.000
*APOE* genotype								
E2/E3	64	188 ± 60	56	93 ± 34^j^	63	46 ± 14	64	263 ± 350^l^
E3/E3	365	193 ± 47	333	109 ± 37^k^	366	49 ± 16	366	186 ± 128^m^
E3/E4	119	198 ± 47	112	112 ± 38^k^	119	48 ± 17	119	195 ± 153^m^
*P* value		0.209		0.002^¶^		0.371		0.033^∗§^
*P* value corrected**		0.836		0.008		1.000		0.132
*LDLR* intron 19G>T (rs6511720)								
G/G	475	193 ± 48	430	107 ± 36	476	48 ± 16	473	198 ± 184
G/T + T/T	114	191 ± 51	107	105 ± 41	113	50 ± 16	114	183 ± 109
*P* value		0.612		0.427		0.120		0.971*
*P* value corrected**		1.000		1.000		1.000		1.000
*SCAP* 2386A>G (rs12487736)								
A/A	164	195 ± 49	146	107 ± 39	164	50 ± 17^n^	164	201 ± 162
A/G	287	194 ± 48	263	107 ± 35	287	49 ± 15^no^	287	197 ± 197
G/G	137	188 ± 47	129	107 ± 41	137	45 ± 14^o^	138	188 ± 124
*P* value		0.443		0.970		0.032^¥^		0.971*
*P* value corrected**		1.000		1.000		0.128		1.000

HDL-C: high-density lipoprotein; LDL-C: low density lipoprotein.

*P value expressed with tests performed with logn-transformed variable.

**P value after Bonferroni correction for multiple testing.

^§^Adjusted for gender, age, and lipid-lowering agents use.

^¶^Adjusted for age, and lipid-lowering agents use.

^$^Adjusted for gender, cigarette smoking, and PI use.

^*£*^Adjusted for gender, age, lipid-lowering agents use, and PI.

^#^Adjusted for gender, age, ethnic group, BMI, and lipid-lowering agents use.

^¥^Adjusted for gender, cigarette smoking, ethnic group, and lipid-lowering agents use.

^ab^Tukey test, *Ins/Ins* versus *Ins/Del*, *P* = 0.619; *Ins/Ins* versus *Del/Del*, *P* = 0.027; *Ins/Del* versus *Del/Del*, *P* = 0.145.

^cd^Tukey test, *Ins/Ins* versus *Ins/Del*, *P* = 0.231; *Ins/Ins* versus *Del/Del*, *P* = 0.006; *Ins/Del* versus *Del/Del*, *P* = 0.071.

^ef^Tukey test, C/C versus C/T, *P* = 0.053; C/C versus T/T, *P* = 0.007; C/T versus T/T, *P* = 0.598.

^ghi^Tukey test, C/C versus C/T, *P* = 0.006; C/C versus T/T, *P* = 0.008; C/T versus T/T, *P* = 0.830.

^jk^Tukey test, E2/E3 versus E3/E3, *P* = 0.011; E2/E3 versus E3/E4, *P* = 0.005; E3/E3 versus E3/E4, *P* = 0.628.

^lm^Tukey test, E2/E3 versus E3/E3, *P* = 0.003; E2/E3 versus E3/E4, *P* = 0.029; E3/E3 versus E3/E4, *P* = 0.875.

^no^Tukey test, A/A versus A/G, *P* = 0.594; A/A versus G/G, *P* = 0.014; A/G versus G/G, *P* = 0.071.

**Table 4:** Effect of *APOB* Del T haplotype in total cholesterol and LDL-C.

Risk haplotype	TC (mg/dL)			LDL-C (mg/dL)		
	*N*	Mean ± SD	*P* value	*N*	Mean ± SD	*P*-value
Noncarriers	213	188 ± 42^a^	0.036	196	100 ± 32^b^	0.011
Heterozygous *Del* T	163	195 ± 51		147	108 ± 36	
Homozygotes* Del* T	27	212 ± 48^a^		25	118 ± 43^b^	

TC: total cholesterol; LDL-C: low density lipoprotein.

Results from analysis of variance.

^a^Tukey test noncarriers versus homozygous for *Del* T, *P* = 0.036.

^b^Tukey test noncarriers versus homozygous for *Del* T, *P* = 0.038.
